# Supplementary material for: Flexoelectric Polarization Enhancement in Paraelectric BaHfO3 via Strain Gradient Engineering
Source: Small. 2025 Dec 16;22(4):e07756. doi: 10.1002/smll.202507756 (PMC12809195; doi:10.1002/smll.202507756)
Supplement: Supplementary file 1 — Supporting Information [file SMLL-22-e07756-s001.docx]

**Supporting Information**

**Flexoelectric Polarization Enhancement in Paraelectric BaHfO_3_ via Strain Gradient Engineering**

***Timo Piecuch^1,2^, Nina Daneu^3^, Jeffrey A. Brock^1,4^, Xiaochun Huang^1,2^,*** ***Tina Radoševič^3^,*** *Arnold M. Müller^5^, Christof Vockenhuber^5^****, Christof W. Schneider^1^, Thomas Lippert^1,2^, Nick A. Shepelin^1^****

**^1^Center for Neutron and Muon Sciences, Paul Scherrer Institute, 5232 Villigen PSI, Switzerland**

**^2^Laboratory of Inorganic Chemistry, Department of Chemistry and Applied Biosciences, ETH Zürich, 8093 Zurich, Switzerland**

**^3^Advanced Materials Department, Jožef Stefan Institute, 1000 Ljubljana, Slovenia**

^4^Laboratory for Mesoscopic Systems, Department of Materials, ETH Zürich, 8093 Zurich, Switzerland

**^5^Laboratory of Ion Beam Physics, ETH Zürich, 8093, Zürich, Switzerland**

**S1: Growth Quality**


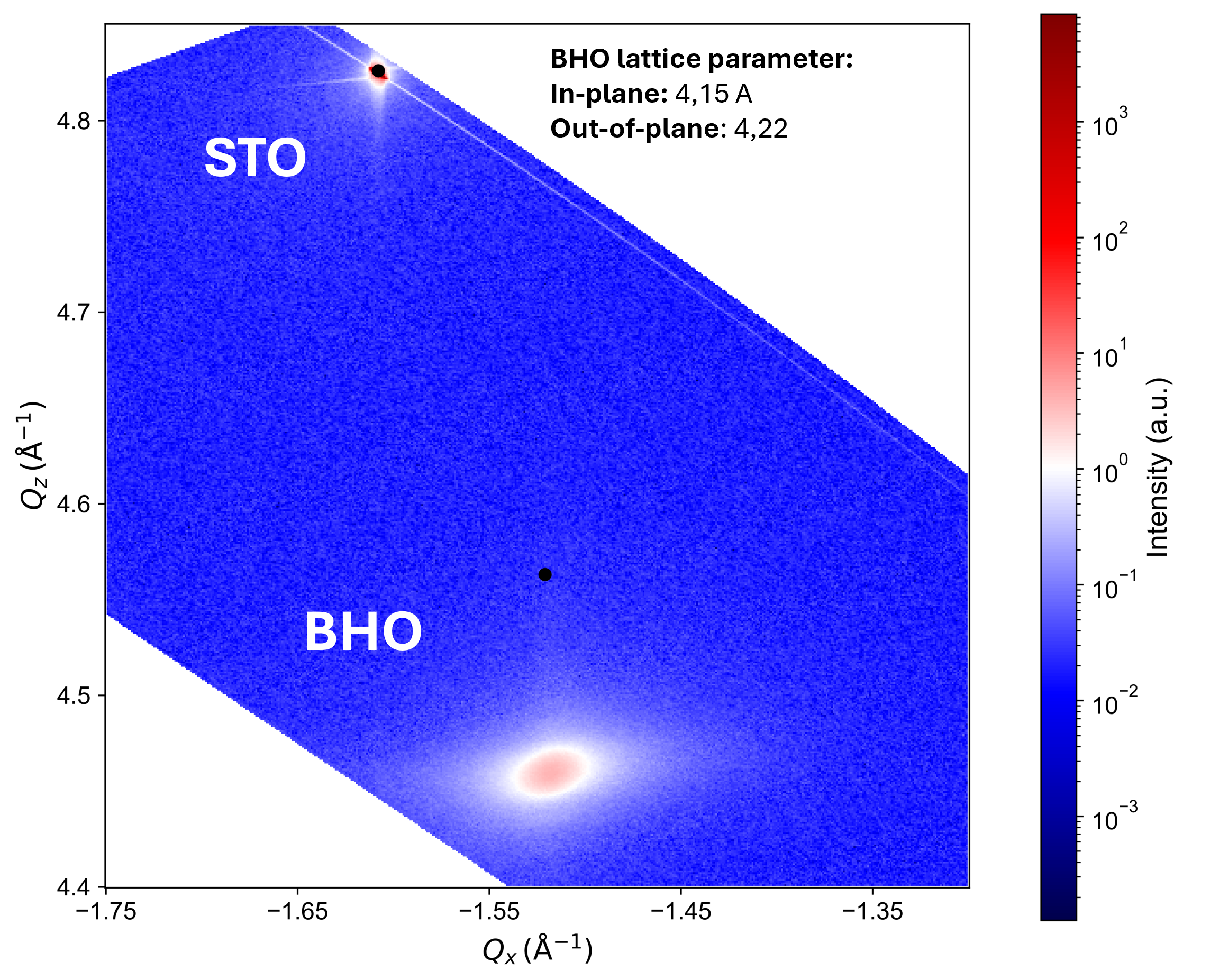


**Figure S1**. RSM of the asymmetrical (-103) signal of BHO directly grown on the STO substrate. The black dots indicate the literature positions of STO and BHO bulk materials. BHO is fully relaxed in-plane, due to a lattice mismatch of 6.8 % between substrate and film and the lack of overlap between the two peaks in Q_x_. The in-plane and out-of-plane lattice parameters of BHO are indicated.





**Figure S2.** XRD 2θ line-scan of the 50 nm thick BHO grown on STO. The inset shows the rocking curve for the film and the substrate. Film full width at half maximum (FWHM) and growth parameters are indicated.


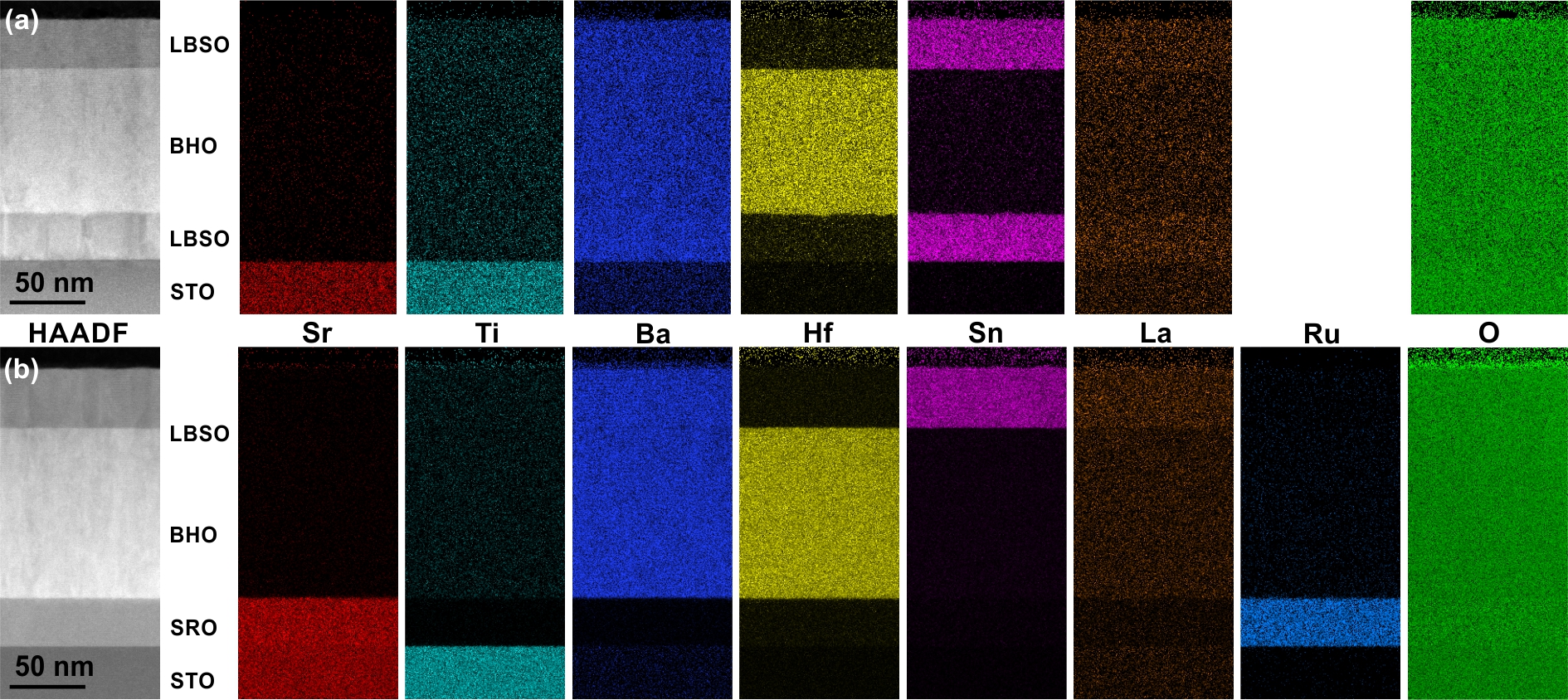
**Figure S4.** Cross-sectional TEM analysis of the capacitor structures: **(a)** STO/LBSO/BHO/LBSO and **(b)** STO/SRO/BHO/LBSO, including elemental mapping.


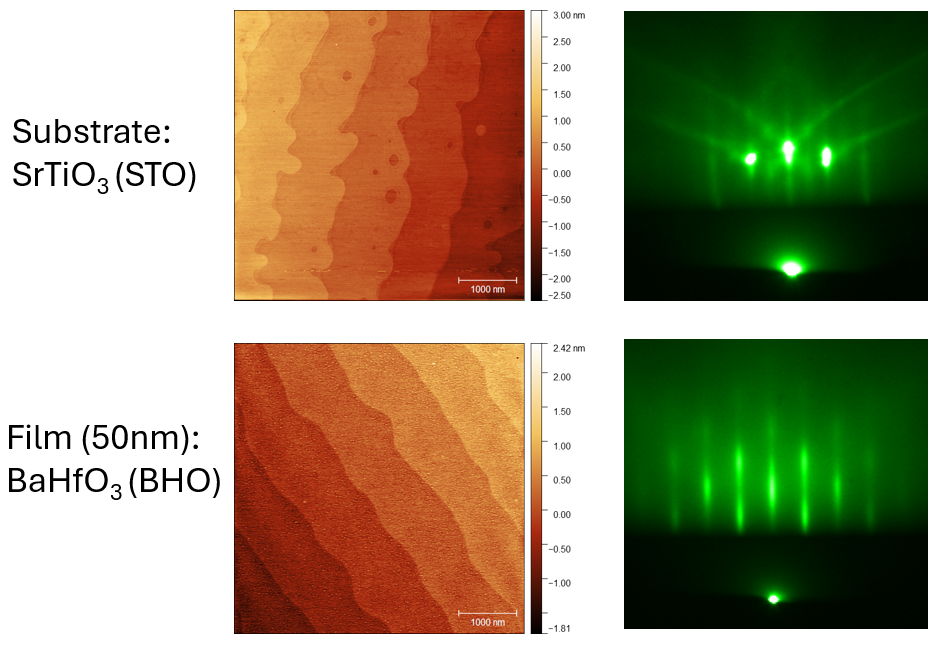


**Figure S3. (left)** AFM topography (5 × 5 µm) and **(right)** RHEED pattern before **(top)** and after **(bottom)** deposition of the 50 nm BHO film on the STO substrate employing the best growth conditions. The AFM data displays evenly spaced terrace steps, and the RHEED pattern shows streaks, both confirming layer-by-layer growth.


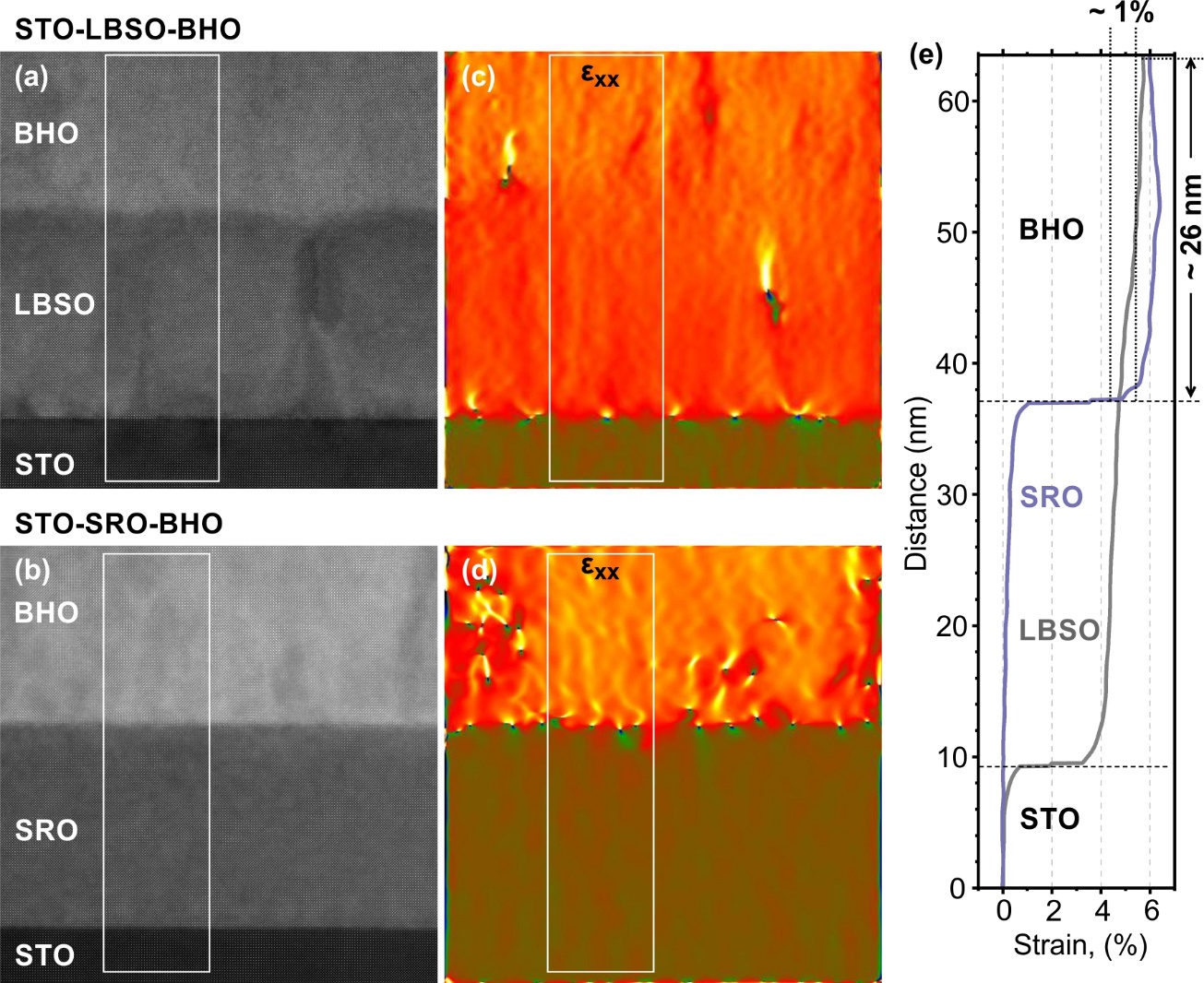
 **Figure S5.** Atomic-resolution micrographs of the **(a)** STO/LSBO/BHO and **(b)** STO/SRO/BHO systems for geometric phase analysis (GPA). **(c,d)** GPA analysis reveals misfit dislocations at the different interfaces and also inside the layers. In addition, the maps were used for quantification of strain relaxation in the in-plane (ε_xx_) direction. The graph in **(e)** shows that the strain in the STO/SRO/BHO system is completely released at the SRO/BHO interface, whereas gradual strain relaxation is observed in the STO/LSBO/BHO system from the STO/LBSO interface onwards. The strain gradient in approximately first 26 nm of the BHO functional layer was calculated based on the relative change of strain per thickness. In this case, strain relaxes for about 1% in the 26 nm, which yields strain gradient of about 3.9 • 10^5^ m^-1^.


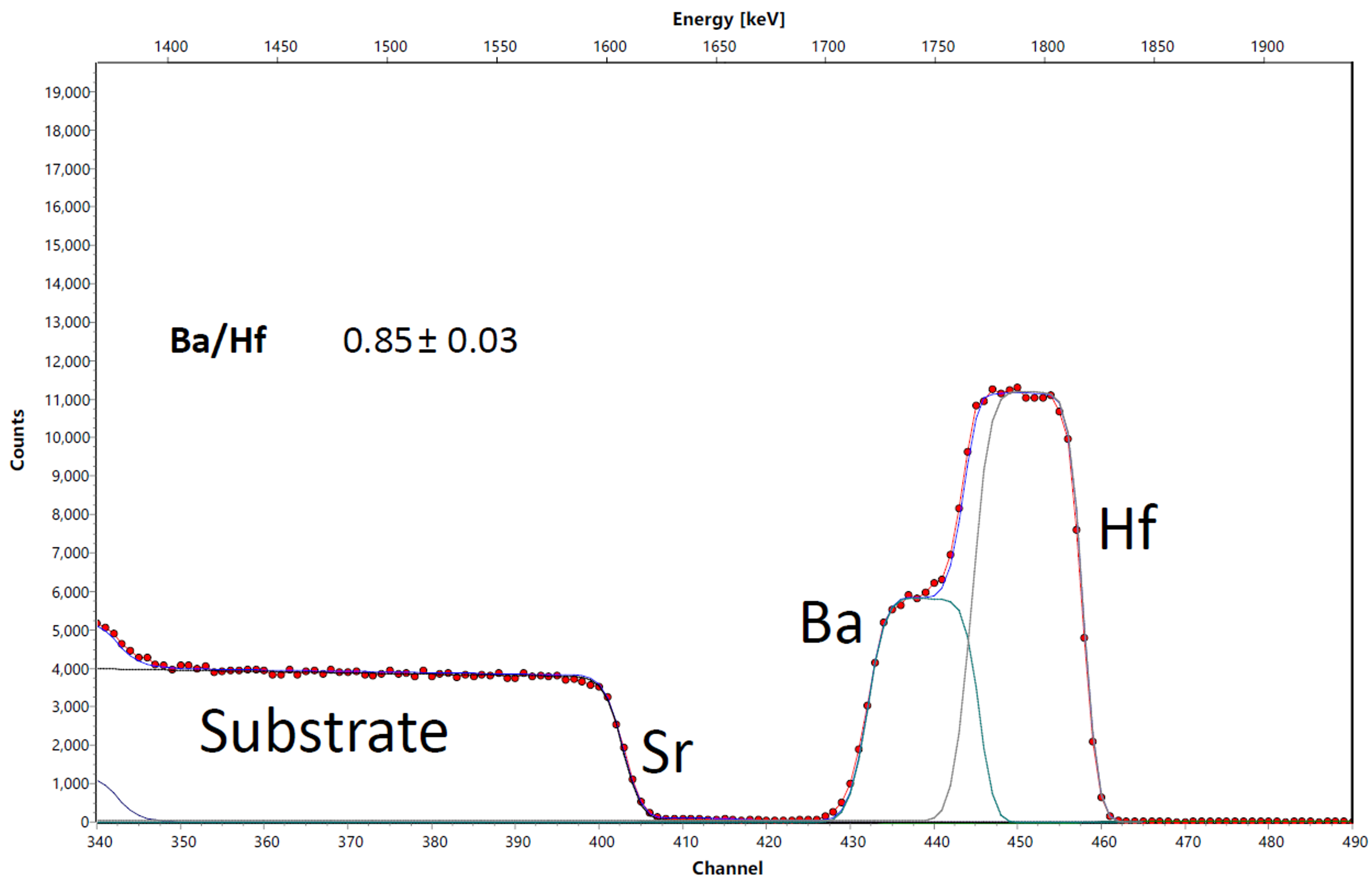


**Figure S6.** RBS spectra of the 50 nm BHO film grown on the STO substrate. Elemental cation ratio is indicated, exposing a Ba deficiency.

**S2: Electrical Properties**


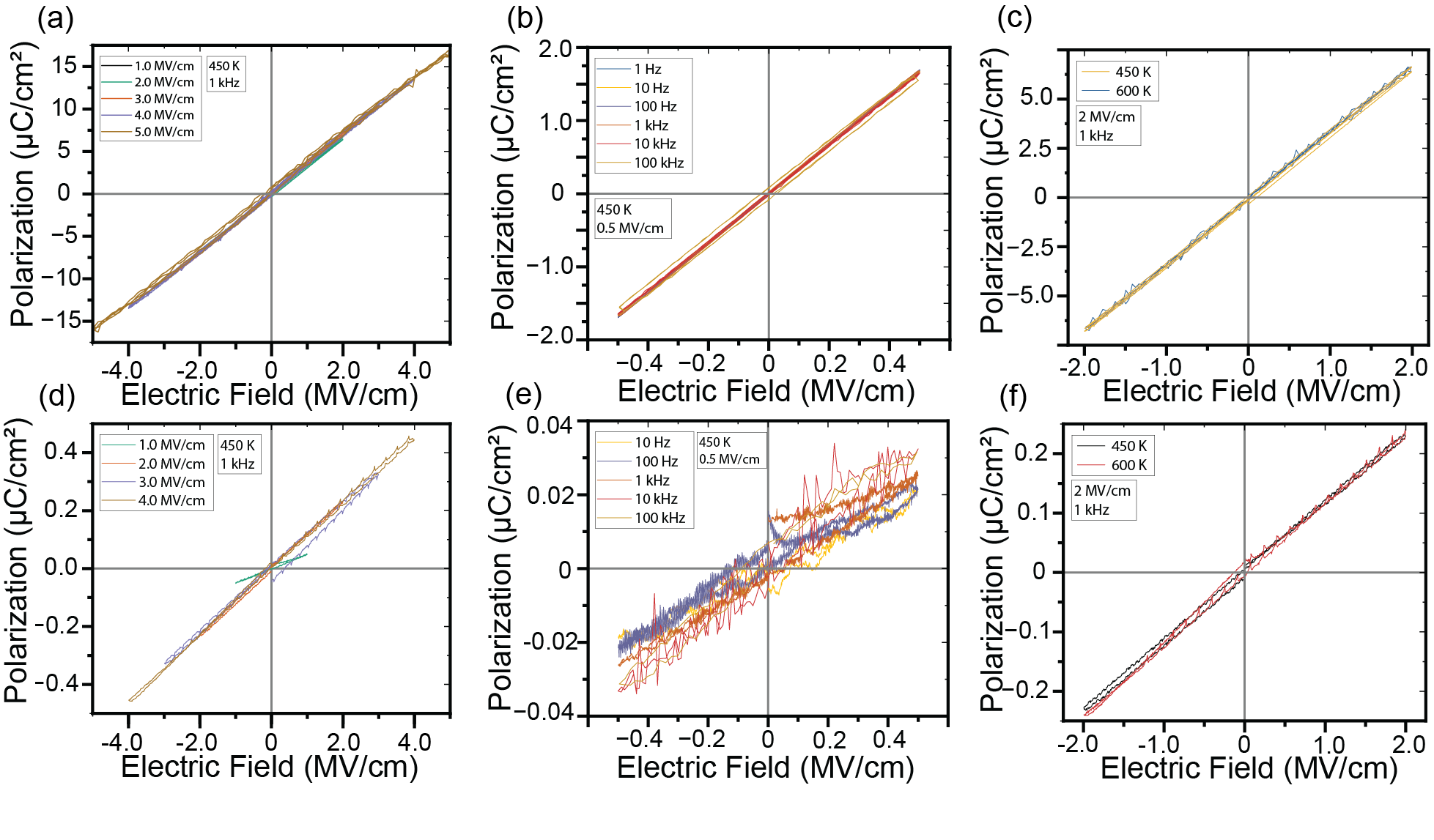


**Figure S7.** P-E hysteresis for the STO – SRO – BHO – SRO structure (**a-c)** and the STO – LBSO – BHO – LBSO structure **(d-f)**. The measurements have been performed for different fields **(a,d)**, different frequencies **(b,e)** and different temperatures **(c,f)**.
